# Independent absorbed-dose calculation using the Monte Carlo algorithm in volumetric modulated arc therapy

**DOI:** 10.1186/1748-717X-9-75

**Published:** 2014-03-14

**Authors:** Akihiro Haga, Taiki Magome, Shigeharu Takenaka, Toshikazu Imae, Akira Sakumi, Akihiro Nomoto, Hiroshi Igaki, Kenshiro Shiraishi, Hideomi Yamashita, Kuni Ohtomo, Keiichi Nakagawa

**Affiliations:** 1 Department of Radiology, The University of Tokyo Hospital, Tokyo, Japan

## Abstract

**Purpose:**

To report the result of independent absorbed-dose calculations based on a Monte Carlo (MC) algorithm in volumetric modulated arc therapy (VMAT) for various treatment sites.

**Methods and materials:**

All treatment plans were created by the superposition/convolution (SC) algorithm of SmartArc (Pinnacle V9.2, Philips). The beam information was converted into the format of the Monaco V3.3 (Elekta), which uses the X-ray voxel-based MC (XVMC) algorithm. The dose distribution was independently recalculated in the Monaco. The dose for the planning target volume (PTV) and the organ at risk (OAR) were analyzed via comparisons with those of the treatment plan.

Before performing an independent absorbed-dose calculation, the validation was conducted via irradiation from 3 different gantry angles with a 10- × 10-cm^2^ field. For the independent absorbed-dose calculation, 15 patients with cancer (prostate, 5; lung, 5; head and neck, 3; rectal, 1; and esophageal, 1) who were treated with single-arc VMAT were selected. To classify the cause of the dose difference between the Pinnacle and Monaco TPSs, their calculations were also compared with the measurement data.

**Result:**

In validation, the dose in Pinnacle agreed with that in Monaco within 1.5%. The agreement in VMAT calculations between Pinnacle and Monaco using phantoms was exceptional; at the isocenter, the difference was less than 1.5% for all the patients. For independent absorbed-dose calculations, the agreement was also extremely good. For the mean dose for the PTV in particular, the agreement was within 2.0% in all the patients; specifically, no large difference was observed for high-dose regions. Conversely, a significant difference was observed in the mean dose for the OAR. For patients with prostate cancer, the mean rectal dose calculated in Monaco was significantly smaller than that calculated in Pinnacle.

**Conclusions:**

There was no remarkable difference between the SC and XVMC calculations in the high-dose regions. The difference observed in the low-dose regions may have arisen from various causes such as the intrinsic dose deviation in the MC calculation, modeling accuracy, and CT-to-density table used in each planning system It is useful to perform independent absorbed-dose calculations with the MC algorithm in intensity-modulated radiation therapy commissioning.

## Introduction

The dose delivered to a patient can be uncertain for several reasons. Dose calculations in patients using a treatment planning system (TPS) represent a main source of dose uncertainty in addition to patient setup, machine stability, and mechanical error [1,2]. The accuracy of patient dose calculations has been continuously improved by the use of the kernel-based superposition/convolution (SC) algorithm [3,4]. Recently, several commercial vendors released the Monte Carlo (MC) algorithms for photon and/or electron beam treatment planning, with the development of faster codes optimized for radiotherapy calculations and improvements in computer processing [5-8]. Consequently, the accessibility and use of MC treatment planning algorithms may become widespread in the radiotherapy community [9,10].

By explicitly modeling the structure of the accelerator, MC algorithms are most credible for determining the absorbed dose, especially in the presence of heterogeneous tissue densities. This may motivate us to use an independently absorbed-dose calculation that is as accurate as the absorbed-dose calculation that was previously verified against commissioning measurements. Unlike the measurement of absorbed doses in phantoms, the independent absorbed-dose calculation using the MC calculation can verify the patient dose, including CT-based heterogeneity correction.

Several studies compared MC calculations with the model-based calculations [11-15]. However, less focus has been directed toward intensity-modulated radiation therapy (IMRT), in which an accurate dose calculation for irregularly shaped segments is required [16,17]. In principle, the added gains in accuracy for MC should be similar for volumetric modulated arc therapy (VMAT) and a multi-field IMRT plan. Because of its dynamical features, it is still useful to use the MC calculation for VMAT verification to identify the cause of the beam delivery error. Almost all previous studies on VMAT verification using MC calculations have been performed for a phantom study or a specific site, and these studies have been limited to the RapidArc system using the analytical anisotropic algorithm (Varian) [18-21]. In this study, we present the result of 15 independent absorbed-dose calculations for various treatment sites in radiation therapy using Elekta VMAT created by a Pinnacle ver. 9.2 TPS (Philips). An X-ray voxel-based MC (XVMC) calculation implemented in the Monaco TPS (Elekta) was used for independent calculations. Sharing common plans, dose volume histograms (DVHs) were analyzed to quantify the dose to the targets and organ at risk (OAR). To classify the cause of the dose difference between the SC and XVMC algorithms, the calculations were compared with the dose measurement data obtained using phantoms.

## Materials and methods

### Ethical consideration and consent

The use of the radiotherapy database for comprehensive and retrospective research has been approved by the committee of the Ethical Review Board in the University of Tokyo Hospital (No. 3372). This research was performed with prior written informed consent. The data was transferred into anonymous one. It makes a definite promise not to use any purpose except this research and to rigid information control.

### Patients and VMAT planning in Pinnacle

Fifteen examples of the clinical treatment for patients with prostate (5 patients), lung (5 patients), head and neck (H&N; 3 patients), rectal (1 patient), or esophageal (1 patient) cancer are presented in this study. All treatment plans were created by SmartArc in Pinnacle v9.2 (Philips, USA) with a 6-MV photon beam of a Synergy linear accelerator (Elekta) using a single arc from −179 to +179 degrees (clockwise) and *D*_95_ prescription for the planning target volume (PTV). For the patients with prostate, H&N, or esophageal cancer, 2 Gy/fraction was prescribed, whereas 2.2 and 12.5 Gy/fraction were prescribed for the patients with rectal and lung cancers, respectively.

A control point (CP) was placed every 2 degrees, resulting in 180 CPs in a single-arc VMAT plan. The final dose calculation was performed with a grid size of 2 mm. The collimator angle was static during rotation. The constraint on multileaf collimator (MLC) motion of 1 mm/degree was applied in the VMAT inverse plan for the patients with lung cancer to avoid moving the MLCs drastically during irradiation in accordance with the protocol for moving targets in our hospital. On the contrary, no constraint on MLC motion was applied for the other patients.

### Independent absorbed-dose calculation in Monaco

The independent absorbed-dose calculation was performed in a Monaco ver. 3.3 TPS using the XVMC code coupled with the virtual source model (VSM) ver. 1.6 based on the virtual energy fluence model of a treatment head including the primary collimator, jaws, and MLC [22,23]. The VSM was created by the vendor (Elekta) using separate internal modeling tools with numerous measurement data instead of MC transport through the detailed linac head components. After treatment planning in Pinnacle, the created plan, patient CT, and regions of interest (ROIs) were transferred to Monaco. At the time of transfer, the VMAT plan was converted into a set of static beams in accordance with Monaco’s format of the patient QA mode by using an in-house program. In the conversion of a VMAT plan with 180 CPs, we divided the static beams into the left (from −359 to −179 degrees) and right halves (from 1 to 179 degrees) because the patient QA mode in Monaco accepts a maximum of 100 beams. In addition, the monitor unit in each beam was multiplied by 10 to avoid a rounding error in the dose calculation in Monaco. The calculated 2-dose distributions were summed by the built-in function in Monaco TPS. The independent absorbed-dose calculation was performed with a grid size of 2 mm and a variance of 0.5% in the total beams.

### Evaluation

**Figure 1:**
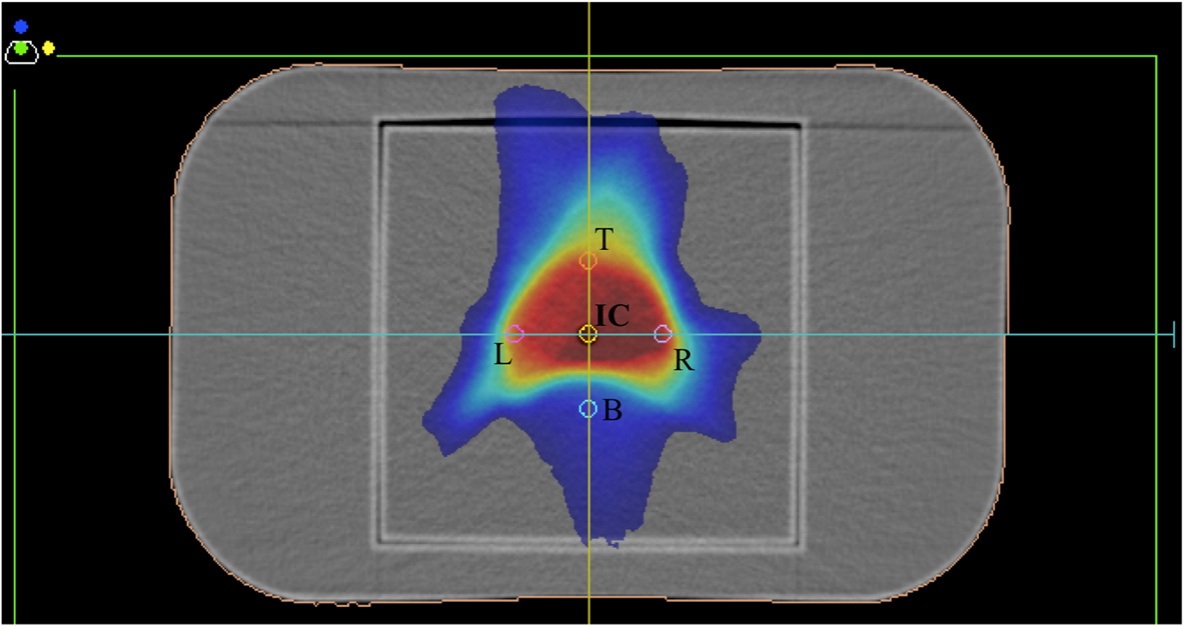
**Computed tomographic image of the water phantom (RT-3000-New-Water, R-Tech, Japan) mapping a dose distribution.** This phantom permits the insertion of a 0.6-mL ionization chamber at the locations indicated as (isocenter), and 3 cm to the left and right and 3 cm above and below the isocenter.

The validation of the beam modeling for both TPSs was performed by comparing the doses for a 10- × 10-cm^2^ field irradiated from 3 different gantry angles (0, 90, and 180 degrees) using a homogeneous water phantom (RT-3000-New-Water, R-Tech, Japan) and a 0.6-mL ionization chamber (Type 30013, PTW, Germany). The locations of the measured volume were the isocenter (IC) and 3 cm to the left (L), 3 cm below (B), 3 cm to the right (R), and 3 cm above (T) the IC (Figure 1). This 3-cm offset provides at least 1 location at which the dose inside the ion chamber is homogeneous (meaning a relatively small standard deviation as presented in Table 1) or corresponds to that in the avoided region (e.g., the bottom in Table 1 for patients with prostate cancer is located in the rectal region).

**Table 1:** Patient-specific doses calculated using the phantoms for the 15 patients

	**Location**	**Pin. (cGy)**	**Mon. (cGy)**	**Meas. (cGy)**	**Pin/Mon**^**1**^	**Pin/Meas**^**2**^	**Mon/Meas**^**3**^	**SD**
Prostate 1	IC	204.6	206.6	208.5	**1.00**	**−1.89**	**−0.91**	0.4
	Left	186.3	185.5	193.1	**−0.44**	**−3.55**	**−3.98**	3.9
	Bottom	107.0	107.7	102.0	**0.67**	**4.86**	**5.57**	21.0
	Right	191.1	189.4	186.4	**−0.93**	**2.56**	**1.61**	3.8
	Top	162.7	162.1	166.8	**−0.36**	**−2.44**	**−2.79**	8.2
Prostate 2	IC	200.5	201.0	198.9	**0.25**	**0.83**	**1.08**	0.4
	Left	184.8	186.4	181.2	**0.87**	**2.01**	**2.90**	2.0
	Bottom	126.5	118.2	126.1	**−6.56**	**0.30**	**−6.28**	31.6
	Right	187.6	189.9	182.4	**1.23**	**2.86**	**4.12**	2.4
	Top	159.8	162.9	150.7	**1.94**	**6.03**	**8.09**	9.3
Prostate 3	IC	198.2	197.1	196.8	**−0.55**	**0.73**	**0.17**	0.4
	Left	175.6	174.7	177.5	**−0.51**	**−1.08**	**−1.59**	4.7
	Bottom	87.4	86.2	86.5	**−1.37**	**1.05**	**−0.34**	4.0
	Right	189.7	190.7	185.4	**0.53**	**2.32**	**2.86**	3.8
	Top	177.5	176.9	175.8	**−0.34**	**0.99**	**0.65**	3.1
Prostate 4	IC	207.9	210.6	208.0	**1.30**	**−0.05**	**1.25**	0.2
	Left	206.1	208.1	204.6	**0.97**	**0.75**	**1.73**	1.0
	Bottom	71.9	70.0	70.8	**−2.64**	**1.53**	**−1.15**	19.5
	Right	204.5	206.7	201.5	**1.08**	**1.47**	**2.56**	1.7
	Top	188.1	190.9	180.5	**1.49**	**4.18**	**5.73**	6.5
Prostate 5	IC	215.4	216.0	218.7	**0.28**	**−1.51**	**−1.24**	0.8
	Left	201.1	201.2	207.5	**0.05**	**−3.10**	**−3.06**	2.3
	Bottom	99.5	97.2	96.3	**−2.31**	**3.36**	**0.97**	30.4
	Right	208.4	208.1	205.1	**−0.14**	**1.62**	**1.47**	2.4
	Top	175.1	175.6	180.4	**0.29**	**−2.94**	**−2.66**	8.6
H&N 1	IC	185.3	186.1	181.4	**0.43**	**2.17**	**2.61**	1.5
	Left	171.9	164.9	167.1	**−4.24**	**2.85**	**−1.34**	1.7
	Bottom	180.5	178.6	173.6	**−1.06**	**3.99**	**2.90**	6.2
	Right	184.9	186.0	177.5	**0.59**	**4.16**	**4.78**	6.4
	Top	171.4	171.9	166.1	**0.29**	**3.17**	**3.47**	3.9
H&N 2	IC	170.9	169.7	165.4	**−0.71**	**3.30**	**2.58**	3.1
	Left	175.2	175.7	168.8	**0.28**	**3.80**	**4.10**	3.5
	Bottom	81.0	79.6	77.4	**−1.76**	**4.60**	**2.79**	4.8
	Right	171.4	173.3	171.3	**1.10**	**0.03**	**1.14**	0.9
	Top	166.4	167.2	161.9	**0.48**	**2.79**	**3.28**	3.2
H&N 3	IC	181.4	183.8	183.0	**1.31**	**−0.90**	**0.41**	1.0
	Left	124.9	123.4	133.8	**−1.22**	**−6.65**	**−7.77**	11.3
	Bottom	177.9	178.6	178.5	**0.39**	**−0.34**	**0.05**	2.8
	Right	142.3	143.5	127.6	**0.84**	**11.55**	**12.49**	6.3
	Top	163.2	164.0	161.6	**0.49**	**0.97**	**1.46**	2.7
Esophagus 1	IC	177.8	177.0	177.6	**−0.45**	**0.11**	**−0.34**	0.3
	Left	183.8	184.2	183.8	**0.22**	**0.00**	**0.22**	0.6
	Bottom	151.5	152.0	144.6	**0.33**	**4.75**	**5.09**	8.5
	Right	161.4	160.0	156.4	**−0.88**	**3.22**	**2.32**	3.9
	Top	160.5	160.4	158.7	**−0.06**	**1.14**	**1.07**	3.7
Rectum 1	IC	211.7	211.1	211.9	**−0.28**	**−0.10**	**−0.38**	0.9
	Left	221.2	221.4	221.8	**0.09**	**−0.27**	**−0.18**	1.4
	Bottom	197.6	197.4	197.2	**−0.10**	**0.21**	**0.11**	1.2
	Right	205.9	207.1	204.6	**0.58**	**0.65**	**1.23**	1.2
	Top	221.2	215.5	219.3	**−2.65**	**0.87**	**−1.73**	1.4
Lung 1	IC	1172.9	1179.8	1201.4	**0.59**	**−2.37**	**−1.79**	NA
Lung 2	IC	1225.5	1233.6	1232.4	**0.65**	**−0.56**	**0.09**	NA
Lung 3	IC	1304.2	1323.5	1295.5	**1.46**	**0.67**	**2.16**	NA
Lung 4	IC	1249.4	1265.0	1230.1	**1.23**	**1.57**	**2.83**	NA
Lung 5	IC	1272.3	1277.1	1275.5	**0.37**	**−0.25**	**0.12**	NA

Five measurement volumes in the water phantom were compared for the patients with prostate, H&N, esophageal, or rectal cancer, whereas the IC point dose in the cork phantom was compared for the patients with lung cancer. For the use of a 0.6-mL ionization chamber, the standard deviation (SD) of the dose inside the chamber evaluated by Pinnacle is indicated in the last column.

^1^([D_Pinnacle] **−** [D_Monaco])/[D_Pinnacle] × 100.

^2^([D_Pinnacle] **−** [D_Measurement])/[D_Pinnacle] × 100.

^3^([D_Monaco] **−** [D_Measurement])/[D_Monaco] × 100.

**Figure 2:**
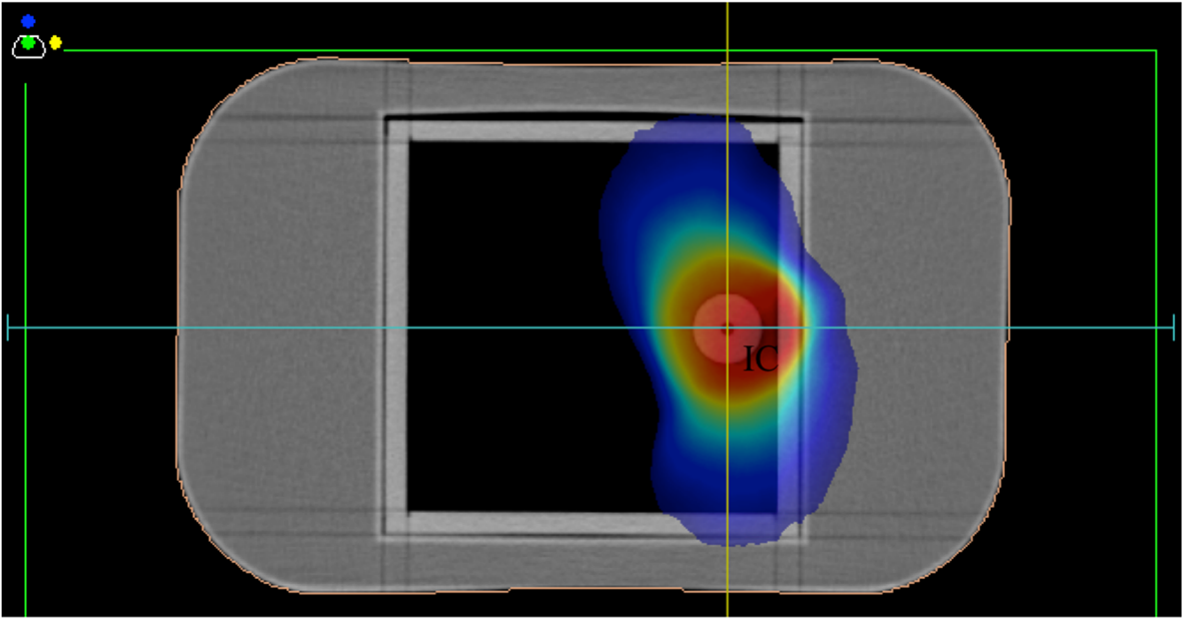
**Computed tomographic image of a cork phantom (RT-3000-New-Water with cork, R-Tech, Japan) mapping a dose distribution.** The low-density area is composed of the piled components of the cork. A spherical insert of 3 cm in diameter is placed anywhere inside the cork, and a 0.015-mL pinpoint ionization chamber can be inserted.

All the treatment plans used in this study were transferred to phantoms, and the absorbed doses were calculated in both TPSs, which were compared with the measurement. The aforementioned water phantom was used for all the treatment plans, excluding those for lung cancer treatment, in which an inhomogeneous cork phantom with a spherical insert of 3 cm in diameter (RT-3000-New-Water with cork, R-Tech) placed on the middle of the cork and a 0.015-mL pinpoint ionization chamber (Type 31014, PTW) were used (Figure 2). Whereas the point dose was employed for comparison with the measurement using the pinpoint chamber, the mean value of the calculated dose inside the ROI for the virtual ionization chamber was used for comparison with the measurement using the 0.6-mL ionization chamber.

In the independent absorbed-dose calculation on the patient CT using Monaco TPS, the following set of clinically relevant dose-volume values per fraction was reported: (1) the minimal, prescription, median, mean, and maximal doses (*D*_min_, *D*_95_, *D*_50_, *D*_mean_, and *D*_max_, respectively) for PTV; (2) the mean and maximal doses for the rectum and bladder of the patients with prostate cancer; (3) the mean and maximal doses for the brainstem and spinal cord of the patients with H&N cancer; (4) the mean dose for both lungs and the contralateral lung of the patients with lung cancer; (5) the mean and maximal doses for the small bowel and bladder of the patients with rectal cancer; and (6) the mean and maximal doses for the spinal cord and the mean dose for the lungs of the patients with esophageal cancer.

To detect systematic differences between the dose calculation algorithms, paired *t* tests were performed for the patients with prostate or lung cancer. A P < 5% was considered statistically significant.

## Results

Table 2 shows the results of the validation test using the water phantom. All the differences between the calculated value and the measurement were within 1.0%. The calculated dose in Monaco TPS was in agreement with those in Pinnacle TPS. Slight differences (more than 1%) were observed at IC and L in the irradiation with a gantry angle of 90 degrees.

**Table 2:** The results of the validation test using a water phantom

	**Location**	**Pin. (cGy)**	**Mon. (cGy)**	**Meas. (cGy)**	**Pin/Mon**^**1**^	**Pin/Meas**^**2**^	**Mon/Meas**^**3**^
Gantry angle	IC	153.0	153.6	153.0	**0.39**	**0.00**	**0.39**
0 degrees	Left	151.7	151.7	151.8	**0.00**	**−0.07**	**−0.07**
200 MU	Bottom	128.9	130.1	129.0	**0.93**	**−0.08**	**0.85**
	Right	151.9	151.7	152.0	**−0.13**	**−0.07**	**−0.20**
	Top	180.8	180.5	180.5	**−0.17**	**0.17**	**0.00**
Gantry angle	IC	119.2	120.8	120.3	**1.34**	**−0.91**	**0.42**
90 degrees	Left	99.7	101.0	100.0	**1.30**	**−0.30**	**1.00**
200 MU	Bottom	118.1	118.1	118.3	**0.00**	**−0.17**	**−0.17**
	Right	142.5	142.9	142.4	**0.28**	**0.07**	**0.35**
	Top	117.8	118.0	118.1	**0.17**	**−0.25**	**−0.08**
Gantry angle	IC	152.0	152.8	152.5	**0.53**	**−0.33**	**0.20**
180 degrees	Left	150.5	150.1	150.3	**−0.27**	**0.13**	**−0.13**
200 MU	Bottom	179.6	180.3	180.0	**0.39**	**−0.22**	**0.17**
	Right	150.3	150.7	150.5	**0.27**	**−0.13**	**0.13**
	Top	128.1	129.2	129.0	**0.86**	**−0.70**	**0.16**

The comparison results are indicated as percentages in last three columns.

^1^([D_Pinnacle] **−** [D_Monaco])/[D_Pinnacle] × 100.

^2^([D_Pinnacle] **−** [D_Measurement])/[D_Pinnacle] × 100.

^3^([D_Monaco] **−** [D_Measurement])/[D_Monaco] × 100.

The results of the patient-specific dose measurement for the 15 patients are shown in Table 1. For all of the patients, excluding those with lung cancer, 5 measurement volumes in the water phantom were compared, whereas for the patients with lung cancer, the IC point dose in the cork phantom was compared. Unlike the validation test, a relatively large difference between the calculated dose and the measurement was observed as presented in Table 1. The main cause of this difference is an inhomogeneous dose inside the 0.6-mL virtual ionization chamber, the standard deviation of which is indicated in the last column in Table 1. We can observe that a large standard deviation, which means that the phantom location is very sensitive for the measurement, produces a relatively large difference between the calculated dose and the measurement, indicating that the measurement volume resulting in a homogeneous dose was in good agreement with the calculation. For patients with lung cancer, the patient-specific dose measurement was acceptable with the point-dose comparison. From the patient-specific QA, again it can be said that the calculated dose in Monaco TPS is in good agreement with those in Pinnacle TPS.

**Figure 3:**
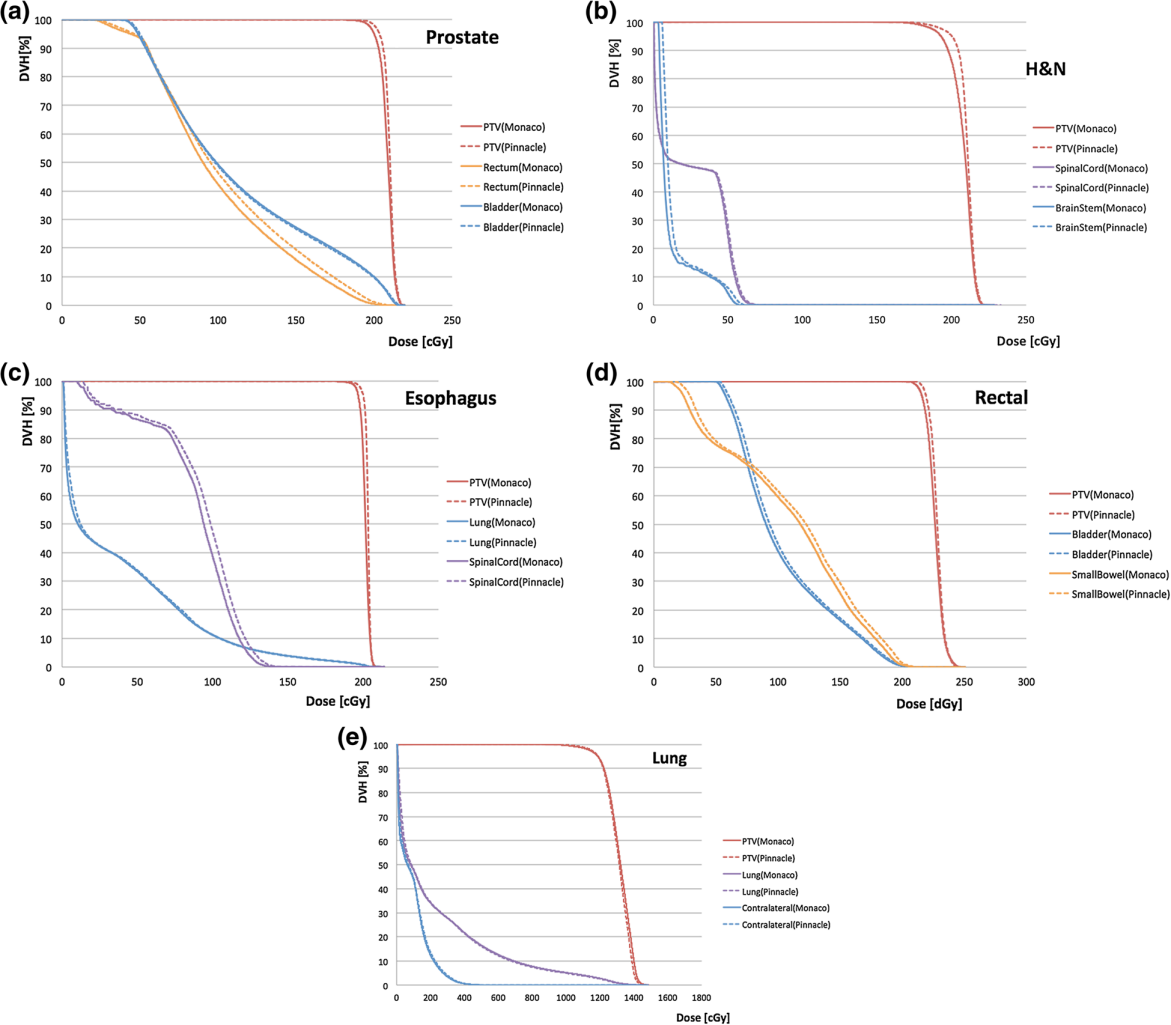
**Dose-volume histogram (1 fraction) for the patients with (a) prostate (No. 1), (b) H&N (No. 1), (c) esophageal, (d) rectal, or (e) lung (No. 1) cancer.** The solid curves denote the Monaco DVH, whereas the dashed curves denote the Pinnacle DVH.

In Table 3, the results of the independent absorbed-dose calculations for the 15 patients are summarized. There were no significant differences between the dose-volume values calculated in Monaco and Pinnacle for PTV. By contrast, a difference was observed in the mean dose for the OAR. For the patients with prostate cancer, the mean rectal dose calculated in Monaco was significantly smaller than that calculated in Pinnacle (P = 0.002). No significant difference was observed for the patients with lung cancer. The examples of DVH for the patients with prostate, H&N, esophageal, rectal, and lung cancer are shown in Figure 3(a), (b), (c), (d), and (e), respectively.

**Table 3:** Difference between the Pinnacle SC and Monaco XVMC calculations regarding the patient CT in percentage ([D_Pinnacle] − [D_Monaco])/[D_Pinnacle] × 100)

**Prostate**	**PTV**					**Rectum**		**Bladder**	
	***D***_**max**_	***D***_**95**_	***D***_**50**_	***D***_**mean**_	***D***_**min**_	***D***_**max**_	***D***_**mean**_	***D***_**max**_	***D***_**mean**_
1	0.30	−1.38	−0.64	−0.73	−4.98	−0.56	−4.59	−0.38	0.25
2	3.31	1.72	0.84	0.94	−2.76	1.37	−4.74	3.67	5.09
3	−0.33	−0.61	−0.37	−0.58	−9.09	−1.00	−4.85	0.92	1.04
4	2.79	1.36	1.41	1.17	1.07	1.82	−3.18	1.73	−5.73
5	0.67	−0.05	0.00	−0.12	−1.42	1.19	−4.59	0.67	1.18
Mean	1.35	0.21	0.25	0.14	−3.44	0.56	−4.39	1.32	0.37
**H&N**	**PTV**					**Spinal cord**		**Brain stem**	
	***D***_**max**_	***D***_**95**_	***D***_**50**_	***D***_**mean**_	***D***_**min**_	***D***_**max**_	***D***_**mean**_	***D***_**max**_	***D***_**mean**_
1	1.15	−2.99	−0.78	−1.28	−6.26	−6.31	−11.70	−3.56	−21.24
2	−0.30	−1.20	−1.94	−1.99	−7.27	0.22	−22.80	0.02	1.79
3	1.60	1.69	2.03	1.83	−1.94	−3.67	−5.77	−1.62	−21.19
Mean	0.82	−0.83	−0.23	−0.48	−5.16	−3.25	−13.42	−1.72	−13.55
**Esophagus**	**PTV**					**Spinal cord**		**Lung**	
	***D***_**max**_	***D***_**95**_	***D***_**50**_	***D***_**mean**_	***D***_**min**_	***D***_**max**_	***D***_**mean**_	***D***_**mean**_	
1	1.07	−0.90	−0.88	−0.85	−1.95	−5.68	−4.74	−2.03	
**Rectum**	**PTV**					**Small bowel**		**Bladder**	
	***D***_**max**_	***D***_**95**_	***D***_**50**_	***D***_**mean**_	***D***_**min**_	***D***_**max**_	***D***_**mean**_	***D***_**max**_	***D***_**mean**_
1	1.76	−0.55	−0.78	−0.78	−0.17	0.62	−2.86	−0.17	−2.54
**Lung**	**PTV**					**PTV-lung**		**Contralateral lung**	
	***D***_**max**_	***D***_**95**_	***D***_**50**_	***D***_**mean**_	***D***_**min**_	***D***_**mean**_		***D***_**mean**_	
1	1.30	−0.10	0.57	0.57	−5.50	−0.29		−4.79	
2	1.21	−0.20	−0.19	−0.12	−1.57	1.85		0.60	
3	−1.34	−0.45	−0.24	−0.34	−0.67	−1.48		5.72	
4	−1.79	−0.03	−0.45	−0.37	0.19	−1.68		−2.14	
5	1.18	−0.70	−0.63	−0.46	−0.74	1.45		0.49	
Mean	0.11	−0.30	−0.19	−0.14	−1.66	−0.03		−0.03	

## Discussion

One of the concerns about the uncertainty in radiation therapy is how well the actual dose administrated to a patient can be reproduced in a TPS, as large deviations can be observed among the different dose calculation algorithms. In this context, the MC algorithm is expected to result in the highest dose calculation accuracy. In our independent absorbed-dose calculation using the XVMC algorithm, it was found that the dose difference in the PTV between the Pinnacle SC and Monaco XVMC algorithms was very small; the agreement in *D*_50_ and *D*_mean_ was within 2.0% in almost all the cases. The agreement for *D*_95_ was also good, but a difference of approximately 3% difference was recorded for patient 1 with H&N cancer (Figure 3[b]). Some differences were observed in *D*_min_, for which the dose profile is likely to be steep; as a result, a slight difference in dose distribution can result in a detectable difference in *D*_min_. In addition, the deviation in the dose calculated using the MC algorithm could affect the differences in *D*_max_ and *D*_min_. The present result confirmed a previous lung IMRT study [17], revealing that a patient dose, including lower attenuation of photon beams such as that observed in lung tissue, is adequately evaluated by the SC algorithm. In VMAT, the various beam path and build-up lengths contribute to the dose in the PTV. Consequently, the dose difference originating from various algorithms would be more moderate than the conventional IMRT when the 3-dimensional density scaling of the kernel is applied for inhomogeneous density regions, such as that used in the Pinnacle TPS. For the lung VMAT, the constraint on the MLC movement described in the Materials and Methods provided a simpler plan than that for the other sites. This finding partially explains why the SC calculation agreed with the MC calculation in the patients with lung cancer.

In contrast to the high-dose regions, a small but significant difference between the Pinnacle and Monaco TPSs was observed in the low-dose regions. As observed in Table 3, the doses for OARs such as the rectum in prostate cancer, the spinal cord in H&N or esophageal cancer, and the small bowel in rectal cancer, as calculated by Pinnacle, tended to be overestimated compared with those calculated by the Monaco XVMC algorithm. In the water phantom study, the difference at B (3 cm below the IC) between Pinnacle and Monaco was similar to that in the patient study. The lower-dose regions are generally more likely to be hidden from irradiation by the MLCs. In this case, the contribution of the primary beam is relatively reduced; consequently, the accuracy of the secondary electron transport is needed in the dose calculation. For this difference, it is also possible to partially resolve this issue by adjusting the MLC transmission and leakage between leaves in either TPS. Therefore, beam remodeling is expected to improve this problem.

In general, the MC calculation is considered more reliable than the SC calculation. However, it should be noted that the MC calculation has an intrinsic deviation arising from statistical accuracy. This uncertainty is larger in low-dose regions. In fact, according to phantom studies, the result of Monaco does not always improve more than that of Pinnacle, although this finding is also influenced by measurement errors. Owing to the low-dose region, the absolute difference was small; from another point of view, it can be said that the dose in Pinnacle agreed with that in Monaco within the limit of measurement accuracy.

A discrepancy in the independent absorbed-dose calculation involves the uncertainty arising from both the beam modeling and CT-to-density curves used in TPS. Pinnacle uses the physical density as the user input, whereas Monaco uses the electron density.

The independent absorbed-dose calculation cannot replace the dosimetric verification performed routinely in patient-specific QA, which also has a role in verifying whether the created treatment plan is actually deliverable in the radiation system. As demonstrated in this study, it would be difficult to elucidate the cause of the dosimetric error from the independent dose calculation even though the MC algorithm was used. However, it does not mean that the independent calculation using the MC algorithm is illogical. The aforementioned statement is based on the assumption that the beam modeling of the TPS is perfect. It is still extremely useful to implement an independent dose calculation as a commissioning from diversified standpoints.

## Conclusions

The independent absorbed-dose calculation indicated that there was no remarkable difference between the SC and XVMC calculations in the high-dose regions. The difference observed in the low-dose regions might have arisen from various causes, including the intrinsic dose deviation in the MC calculation, the modeling accuracy depending on the parameters such as MLC transmission and scattering, and the CT-to-density table used in each TPS. It is meaningful for IMRT commissioning to verify the modeling accuracy.

## Competing interests

K.N. received the research grant from Elekta.

## Authors’ contributions

KN conceived the idea. AH, AS, and AN generated the treatment plan in Pinnacle. AH developed the program and drafted the manuscript. AH, ST, and TI calculated the dose in Monaco. AH and TM analyzed the data. ST and TI conducted the measurement. HI, KS, HY, KO, and KN gave the clinical advice and received the informed consents. All authors read and approved the final manuscript.
